# *Streptococcus pneumoniae* Serotype 1 Capsular Polysaccharide Induces CD8^+^CD28^−^ Regulatory T Lymphocytes by TCR Crosslinking

**DOI:** 10.1371/journal.ppat.1000596

**Published:** 2009-09-25

**Authors:** Janina Mertens, Mario Fabri, Alessandra Zingarelli, Torsten Kubacki, Sonja Meemboor, Laura Groneck, Jens Seeger, Martina Bessler, Helena Hafke, Margarete Odenthal, Joan G. Bieler, Christoph Kalka, Jonathan P. Schneck, Hamid Kashkar, Wiltrud M. Kalka-Moll

**Affiliations:** 1 Institute for Medical Microbiology, Immunology and Hygiene, University of Cologne Medical Center, Köln, Germany; 2 Department of Pathology, University of Cologne Medical Center, Köln, Germany; 3 Swiss Cardiovascular Center, Division of Vascular Medicine, Inselspital, Bern University Hospital, and University of Bern, Freiburgstrasse, Bern, Switzerland; 4 Department of Pathology, Division of Immunopathology, Johns Hopkins School of Medicine, Baltimore, Maryland, United States of America; University of Pennsylvania, United States of America

## Abstract

Zwitterionic capsular polysaccharides (ZPS) of commensal bacteria are characterized by having both positive and negative charged substituents on each repeating unit of a highly repetitive structure that has an α-helix configuration. In this paper we look at the immune response of CD8^+^ T cells to ZPSs. Intraperitoneal application of the ZPS Sp1 from *Streptococcus pneumoniae* serotype 1 induces CD8^+^CD28^−^ T cells in the spleen and peritoneal cavity of WT mice. However, chemically modified Sp1 (mSp1) without the positive charge and resembling common negatively charged polysaccharides fails to induce CD8^+^CD28^−^ T lymphocytes. The Sp1-induced CD8^+^CD28^−^ T lymphocytes are CD122^low^CTLA-4^+^CD39^+^. They synthesize IL-10 and TGF-β. The Sp1-induced CD8^+^CD28^−^ T cells exhibit immunosuppressive properties on CD4^+^ T cells *in vivo* and *in vitro*. Experimental approaches to elucidate the mechanism of CD8^+^ T cell activation by Sp1 demonstrate in a dimeric MHC class I-Ig model that Sp1 induces CD8^+^ T cell activation by enhancing crosslinking of TCR. The expansion of CD8^+^CD28^−^ T cells is independent, of direct antigen-presenting cell/T cell contact and, to the specificity of the T cell receptor (TCR). In CD8^+^CD28^−^ T cells, Sp1 enhances Zap-70 phosphorylation and increasingly involves NF-κB which ultimately results in protection versus apoptosis and cell death and promotes survival and accumulation of the CD8^+^CD28^−^ population. This is the first description of a naturally occurring bacterial antigen that is able to induce suppressive CD8^+^CD28^−^ T lymphocytes *in vivo* and *in vitro*. The underlying mechanism of CD8^+^ T cell activation appears to rely on enhanced TCR crosslinking. The data provides evidence that ZPS of commensal bacteria play an important role in peripheral tolerance mechanisms and the maintenance of the homeostasis of the immune system.

## Author Summary

One of the most difficult challenges for the mammalian immune system is to protect its host from pathogens and cancer while at the same time avoiding a self-destructive or overwhelming immune response. In addition to so-called central tolerance induced in the thymus, the immune system relies on peripheral control mechanisms. One of the most important brakes of the peripheral tolerance system is constituted by so-called regulatory T lymphocytes. The predominately investigated regulatory T lymphocytes belong to the CD4^+^ subset but CD8^+^ regulatory T lymphocytes are now also believed to play a major role in controlling immune responses. Herein, we describe for the first time a natural occurring saccharide antigen from a commensal bacterium which induces the accumulation of a defined population of CD8^+^ regulatory T lymphocytes. These CD8^+^ regulatory lymphocytes suppress inflammatory immune responses *in vivo* and in *in vitro* assays. We also describe how the bacterial antigen induces the activation of CD8^+^ T cells. Our findings not only describe a novel mechanism of saccharide-mediated T cell activation but also provide evidence that commensal bacteria play an important role in the induction of peripheral tolerance and maintenance of the mammalian immune system.

## Introduction

Humans are colonized by multitudes of commensal organisms the importance of which is now recognized for good health. Capsular polysaccharides of the physiologic human bacterial flora are immunogenic components that first encounter the immune system during their initial colonization, and, at the time the immune system is developing and maturing. As opposed to common negative charged polysaccharides, the biologic activity of polysaccharides from certain commensal bacteria is unique in their ability to stimulate CD4^+^ T cells *in vivo* and *in vitro* [1],[2],[3],[4],[5]. Examples of such bacteria are, *Bacteroides fragilis* the ubiquitous anaerobic member of the gut flora, *Staphylococcus aureus* a temporary member of the skin and mucosal flora, and, *Streptococcus pneumoniae* of the upper respiratory tract flora. CD4^+^ T cell activation induced by these polysaccharides depends on their unique electrical charge. Each repeating unit has a minimum of one positive and one negative charge leading to their common three-dimensional configuration characterized by a right-handed helix with repeating negatively charged grooves, the positive charges being on the outer surface of the lateral boundaries [1],[4],[6],[7]. Elimination of their charged groups abrogates T cell-dependent immune responses *in vitro* and *in vivo* [1]. Introduction of a zwitterionic charge motif into a non-ZPS converts a T cell-independent polysaccharide into a CD4^+^ T cell-dependent antigen [1],[8]. Presentation of the so-called zwitterionic polysaccharide (ZPS) from *S. pneumoniae* serotype 1 (Sp1) by MHC class II molecules requires its retrograde transport from lysosomes to the cell surface within tubules as a ZPS/MHC class II complex and also, the DM molecule [9],[10]. Binding studies suggest that ZPS binds to the antigen binding cleft of the MHCII molecule [11]. ZPS directs the development of the systemic cellular immune response by correcting CD4^+^ T cell deficiencies and T_H_1/T_H_2 imbalances towards a T_H_1 immune response [12]. Intraperitoneal application of ZPS, such as PS A1 from *B. fragilis* or Sp1 from *S. pneumoniae*, with adjuvant, promotes CD4^+^ T cell-dependent intra-abdominal abscess pathology [5],[13], whereas subcutaneous ZPS application without adjuvant induces regulatory IL-10-secreting CD4^+^ T cells that protect from abscess formation [14]. Recently, it was shown that oral application of ZPS prevents inflammatory bowel disease (IBD) [15]. The ZPS PS A1 protects from IBD through a functional requirement for interleukin (IL)-10-producing CD4^+^ T cells.

A significant number of potentially self-reactive lymphocytes leave the primary lymphoid organs and are kept under control by “peripheral tolerance mechanisms” [16]. Probably the most important of these mechanisms is assured by regulatory CD4^+^ and CD8^+^ T lymphocytes capable of suppressing adaptive and also innate immune-responses [17],[18]. Regulatory T cells are known to control immune responses to self-antigens but also to nonself-antigens (e. g. upon infection [19],[20]) and to innocuous (probably non-self) antigens in intestinal mucosa [21]. “Naturally” occurring regulatory/suppressive CD8^+^ T lymphocytes (Ts) constitute an endogenous long-lived population of T cells that develop in the thymus [22],[23]. In mice, CD8^+^CD122^+^ T cells have received special attention. As naturally occurring Ts, they are regarded as a functional T cell subset that affects immunity through the release of the anti-inflammatory cytokine IL-10 [24],[25]. They are believed to have specificity for self antigens and are poised to prevent autoimmunity [26]. Mice genetically deficient for CD122 (the IL-2/IL-15 β-receptor chain), spontaneously develop severe hyperimmunity. Furthermore, adoptive transfer of CD8^+^CD122^+^ Ts ameliorates established experimental autoimmune encephalomyelitis [27]. Another population of described Ts is termed “adaptive” or “induced” because these Ts are induced after one time or several rounds of stimulation with cytokines, antibodies, allogeneic or xenogeneic stimulator cells, antigen-pulsed APCs, or antigens mostly of an artificial synthetic peptide (overview in [28]). However, data from *in vitro* induced CD8^+^CD28^−^ Ts after priming with specific antigens suggests that the Ts do not respond to the priming antigens [29]. No natural antigen either self, or tumor, or bacterial, has ever been demonstrated to induce the differentiation of CD8^+^CD28^−^ T cells or any other subset of CD8^+^ Ts *in vitro* or *in vivo*. CD8^+^CD28^−^ Ts were shown to suppress autologous and heterologous CD4^+^ T cell proliferation by rendering APC tolerogenic through the induction of receptors that transmit negative signals. Specifically, upregulation of immunoglobulin-like transcripts (ILT) 3 and ILT4 [30]. CD8^+^CD28^−^ Ts have been attributed to be players in autoimmunity [31]. IL-10 producing CD8^+^CD28^−^ Ts infiltrate tumors, circulate in the peripheral blood of the majority of cancer patients, and inhibit both proliferative and cytotoxic T cell responses in this setting [32]. Moreover, CD8^+^CD28^−^ Ts seem to regulate immune-responses in inflammatory disease [33]. CD8^+^CD28^−^ Ts are also involved in the control of mucosal immune responses. In an IBD model [34], in contrast to CD8^+^CD28^+^, CD8^+^CD28^−^ T cells freshly isolated from the spleen or gut of naïve mice, being rather “natural” Ts, effectively prevent the development of colitis. CD8^+^CD28^−^ T cells derived from IL-10–deficient mice lack the functional ability to prevent colitis. Moreover, IBD induced with colitogenic T cells incapable of responding to TGF-β is not prevented with CD8^+^CD28^−^ Ts. These data suggest that in addition to IL-10, TGF-β also seems to play a role in the suppressive activity of CD8^+^CD28^−^ Ts. Other “naturally” and “adaptive” murine and human Ts populations have also been described [35],[36],[37],[38],[39],[40]. Various cell surface markers such as, CTLA-4, CD25, CD62L, CD122 and the lack of expression of CD28, CD44, the synthesis of the cytokines TGF-β and IL-10, and the expression of Foxp3 have been reported in Ts [28]. Not all cell surface markers are expressed on one subset of Ts, and not all cytokines are produced by one particular Ts subset. It is currently unknown whether the different types of Ts are distinct cell populations, overlapping, or, are essentially derived from one source. They seem to emerge after T cell receptor (TCR) stimulation, and exhibit their down-regulatory function by impairing the responsiveness of other T cells.

TCR is the primary trigger for antigen-mediated T cell stimulation. In principal three different mechanisms of antigen recognition by the TCR are known [41]. Firstly, conventional peptide antigens are loaded onto MHC molecules within the binding groove for antigen recognition by the TCR. Secondly, superantigens polyclonally activate T cells by bridging the outer surface of the MHC and the TCR molecule. Thirdly, mitogens and lectins induce T cell activation by crosslinking T cell surface molecules such as the TCR and CD3.

For all three mechanisms, engagement of the TCR by an antigen translates into an intracellular signal by inducing a phosphorylation cascade resulting in the activation of ζ-associated protein of 70 kDa (Zap-70). Activated Zap-70 phosphorylates scaffold proteins resulting in the activation of transcription factors such as NFκB that initiate gene programs promoting proliferation, differentiation, and effector actions of T cells. TCR- and co-stimulatory CD28-mediated signaling effect the susceptibility to apoptosis.

Previous investigations have exclusively focused on the role of effector and regulatory CD4^+^ T cells in ZPS-mediated immune responses. However, the role of CD8^+^ T cells in ZPS-mediated immunity has not been addressed to date. Here, we analyzed the ability of a ZPS to regulate CD4^+^ T cell immune responses through CD8^+^ T cells. We found that Sp1 induces the expansion of regulatory CD8^+^CD28^−^ T lymphocytes *in vitro* and *in vivo* and we assessed their function and activation mechanism.

## Results

### CD8^+^CD28^−^ T cells modulate Sp1-mediated CD4^+^ T cell-dependent intraperitoneal abscess formation

**Figure 1:**
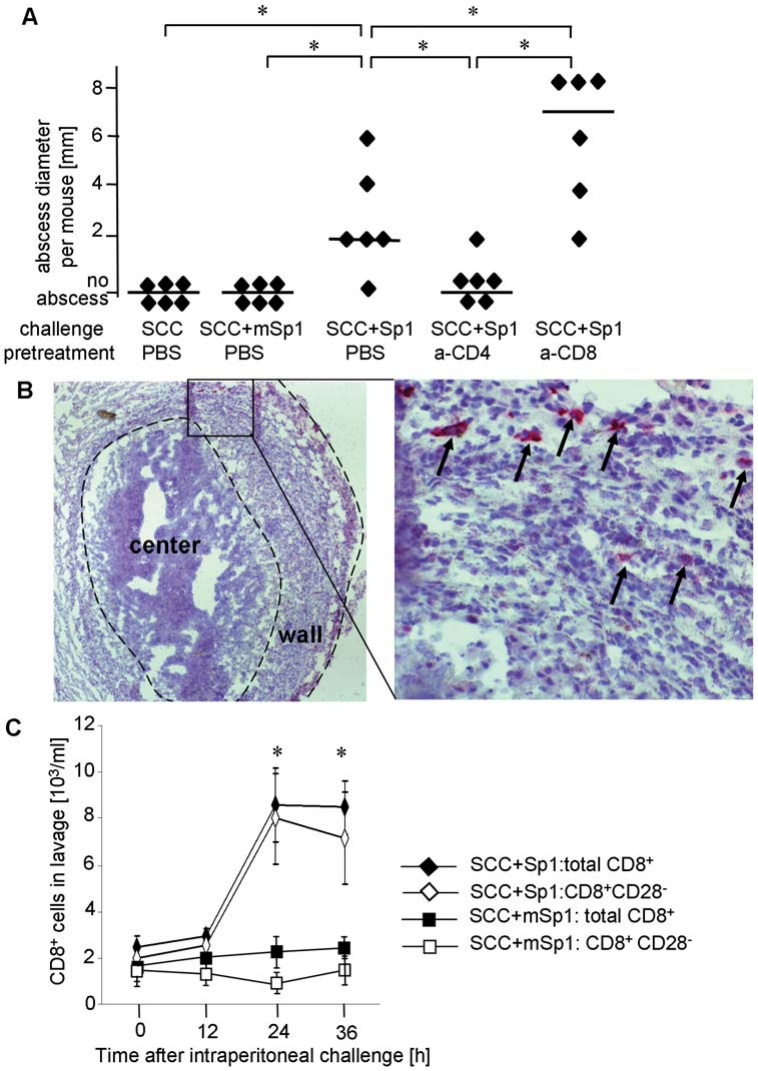
CD8^+^ T cells modulate abscess formation in response to intraperitoneal Sp1-application. A. C57BL/6 WT mice received an anti-CD4-mAb, an anti-CD8-mAb, or PBS intravenously 24 h prior to intraperitoneal Sp1-challenge, or challenge with chemically modified Sp1 (mSp1) plus sterile cecal content (SCC) adjuvant, or SCC alone as controls. After 6 days, examination for intraperitoneal abscess formation was performed at autopsy. Each dot represents the abscess diameter per mouse; bars indicate the median abscess size per group; *p<0.05. The experiment was performed three times independently with 6 mice per group, respectively. One representative experiment is shown in the figure. B. Immunohistochemistry of Sp1-induced abscesses was performed with an anti-CD8-mAb. The left panel shows an overview of an abscess. The right panel shows the magnified detail of the abscess wall within the rectangular box of the left panel. Some CD8^+^ T cells are labeled with black arrows. The figures show one representative result out of three experiments. C. The influx of CD8^+^ and CD8^+^CD28^−^ T cells into the peritoneum was determined in C57BL/6 mice challenged with Sp1 or mSp1 plus SCC as a control. The peritoneal lavage was performed at different time intervals (x-axis) after challenge. Cell numbers were determined by cell counting and flow cytometry. Bars indicate standard deviations within a tested group; *p<0.05. The experiment was performed two times with each 4 mice per group and time point. The figure shows one representative result.

To study CD8^+^ T cell immunity to ZPS, we first employed a well characterized experimental model of CD4^+^ T cell-dependent immune response, which is the mouse model of Sp1-mediated abscess formation [10]. Whole ZPS-positive commensal bacteria or their ZPS, such as Sp1, induce intraperitoneal abscesses when applied intraperitoneally with sterile cecal content (SCC) adjuvant [1]. Application of adjuvant alone and chemically modified Sp1 (mSp1) plus adjuvant, as negative controls, do not induce abscesses (Fig. 1A). In contrast, challenging C57BL/6 WT mice with intraperitoneal Sp1 plus adjuvant results in the formation of sterile abscesses six days later. The median abscess size of Sp1-challenged mice is 2 mm (one abscess/mouse). CD4^+^ T cell-dependency of this immune response is confirmed, as CD4^+^ T cell depletion leads to significant inhibition of abscess formation (no abscesses observed). In contrast, CD8^+^ T cell depletion results in the formation of significantly larger abscesses with a median abscess size of 7 mm (one abscess/mouse). Immunohistochemical analysis of Sp1-mediated intraperitoneal abscesses shows that CD8^+^ T cells participate in the formation of the organized wall of abscesses (Fig. 1B). Intraperitoneal Sp1 challenge results in a significant increase of the total cell count in the peritoneal cavity peaking at 24 h and 36 h following Sp1 challenge [42]. The cellular influx mainly consists of neutrophils and macrophages (not shown). DCs (about 7%) and CD4^+^ T cells (about 1%) also are attracted by Sp1 into the peritoneal cavity [10],[42]. Here, we analyzed the quantity and phenotype of CD8^+^ T cells migrating into the peritoneum after Sp1 application. To establish the distinction of CD28^−^ and CD28^+^ CD8^+^ T cells fluorescent staining of CD28 was performed with native spleen CD8^+^ T cells that contained a mixture of CD28^−^ and CD28^+^ CD8^+^ T cells (data not shown). As no clear CD8^+^CD28^−^ population could be distinguished CD8^+^CD28^−^ cells are therefore defined as those expressing CD28 at background isotype levels. The thus defined CD28^−^ population represents about 30% of CD8^+^ splenocytes as previously described [34]. Flow cytometry analysis of the CD28 phenotype of CD8^+^ T cells migrating into the peritoneal cavity 24 h and 36 h after Sp1 challenge reveals that almost all CD8^+^ T cells are CD8^+^CD28^−^ cells (Fig. 1C). We conclude that Sp1 attracts CD8^+^C28^−^ T cells into the peritoneal cavity that modulate CD4^+^ T cell-dependent abscess formation.

### Sp1 induces CD8^+^CD28^−^ T lymphocytes positive for TGF-β and IL-10

**Figure 2:**
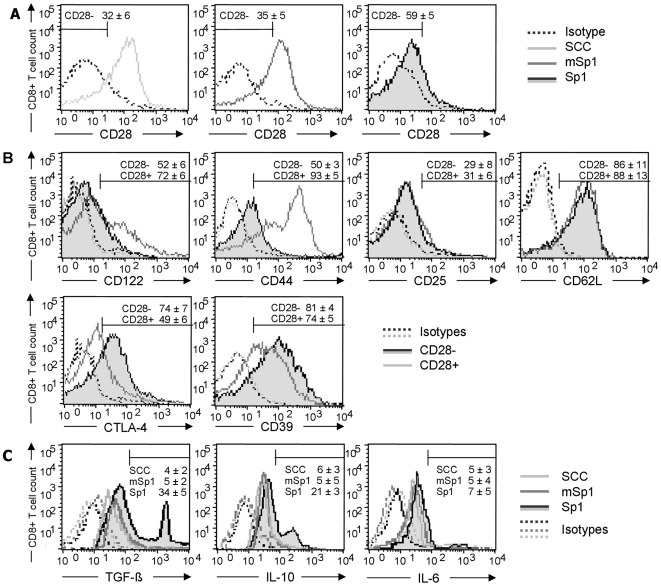
Phenotype of Sp1-induced CD8^+^CD28^−^ T lymphocytes *in vivo*. C57BL/6 WT mice were challenged intraperitoneally with Sp1, mSp1 (100 µg/mouse, respectively), or SCCA alone. After 18 h, spleen cells were isolated. Cells were stained for CD8 and CD28 surface expression and prepared for flow cytometry. Flow cytometry histograms in A–C are representative for one out of five experiments with 4 mice each. A. Intraperitoneal Sp1 application induces CD8^+^CD28^−^ T lymphocytes *in vivo*. The percentages ±SD (n = 4) of the respective CD8^+^CD28^−^ cells are given above the marker. B. For determination of the phenotype of the Sp1-induced CD8^+^CD28^−^ T lymphocytes in C57BL/6 mice, CD8^+^CD28^−^ and CD8^+^CD28^+^ T cells were stained with antibodies specific for surface markers and prepared for flow cytometry analysis. Percentages of positive cells±SD (n = 4) for the CD8^+^CD28^−^ and CD8^+^CD28^+^ T cell subsets are displayed above the marker. C. C57BL/6 WT mice were challenged intraperitoneally with Sp1, mSp1 (100 µg/mouse, respectively), or SCC alone. After 18 h, spleen cells were isolated. CD8^+^CD28^−^ cells were analyzed for LAP surface expression and intracellular cytokines by flow cytometry. Percentages of positive cells ±SD (n = 4) are given below the marker.

To further characterize the phenotype of the Sp1-induced CD8^+^CD28^−^ T lymphocytes, we first investigated whether intraperitoneal Sp1-challenge also induces CD8^+^CD28^−^ T cells in the spleens of C57BL/6WT mice which had been challenged the day before with intraperitoneal Sp1. Compared to SCCA- and mSp1-challenged mice, Sp1 induces an expansion of CD8^+^CD28^−^ T cells by 27% and 24%, respectively (Fig. 2A). Different CD8^+^CD28^−^ T cell subpopulations have been characterized by means of the appearance of the surface markers expressed and secretion of cytokines. Sp1-induced CD8^+^ T cells expressed CD122, albeit mainly at low levels (Fig. 2B). Whereas all CD8^+^CD28^−^ cells were CD122^low^, a fraction of CD8^+^CD28^+^ cells expressed high levels of CD122. To analyze the expression of markers that allow the distinction of naïve, activated, and memory T cells, we stained the Sp1-induced CD8^+^CD28^−^ T cells and CD8^+^CD28^+^ T cells as controls with CD44, CD25, and CD62L (Fig. 2B). Amongst CD28^+^ cells, a population of CD44^high^ activated/memory CD8^+^ T cells was found. In contrast, CD28^−^ cells homogenously expressed CD44 at a low level (50% positive cells) that implies a naïve phenotype. Further phenotyping by determination of the CD25 and CD62L surface markers of Sp1-induced CD8^+^CD28^−^ T cells showed a CD25^low^ and CD62L^high^ pattern which confirms them as naïve quiescent CD8^+^ T cells [34],[43],[44],[45]. We also determined the expression of CTLA-4 and CD39. CD39 is an ectonucleotidase that had been found positive in CD4^+^ regulatory T cells [46]. CD8^+^CD28^−^ T cells show an increased expression of CTLA-4 (22%) and CD39 (19%) when compared to CD8^+^CD28^+^ T lymphocytes.

Immunomodulation by several regulatory T cell populations often involves TGF-β and IL-10. We therefore investigated if CD8^+^CD28^−^ cells can produce these cytokines. CD8^+^CD28^−^ T cells were isolated from spleens of mice challenged the day before with Sp1, and mSp1, or SCC alone as controls. Latency Associated Peptide (LAP) is a proteolytic product of the pro TGF-β1 protein and its surface expression is therefore limited to TGF-β1 expressing cells [47]. As shown in Fig. 2C, in comparison to mice challenged with SCC and mSp1 plus SCC, a substantial proportion (34%) of CD8^+^CD28^−^ cells expressed LAP. We observed that a substantial proportion (21%) of CD8^+^CD28^−^ cells produced IL-10. All groups showed background levels which is likely due to basic cytokine expression caused by the adjuvant SCC. However, the detection of IL-6, that has not been described to play a role in immunosuppressive CD8^+^ T cell and thus was used as a negative control, was low at 5%.

### Sp1-induced CD8^+^CD28^−^ T cells suppress CD4^+^ T cell-dependent immune responses

**Figure 3:**
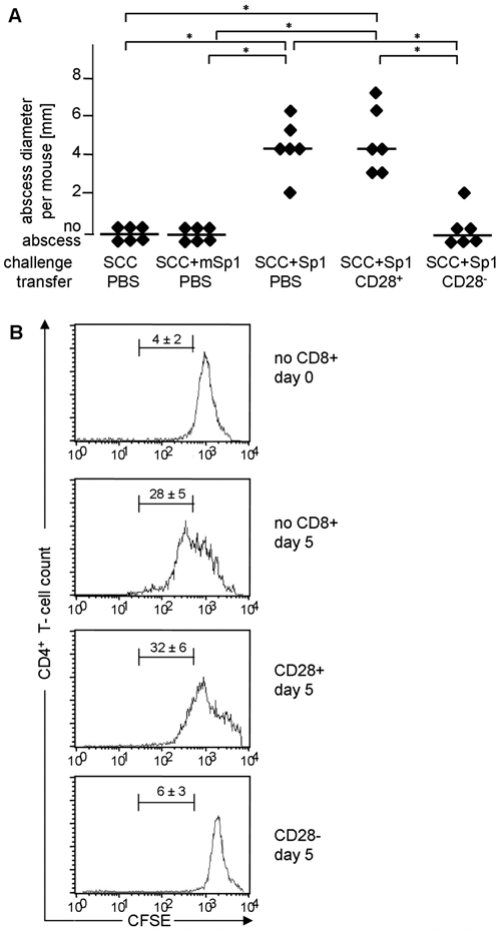
Sp1-induced CD8^+^CD28^−^ T lymphocytes suppress CD4^+^ T cell immune responses *in vivo* and *in vitro*. A. Sp1-induced CD8^+^CD28^−^ T lymphocytes suppress intraperitoneal abscess formation. C57BL/6 mice received intraperitoneal Sp1 challenge (100 µg), and challenge with mSp1 (100 µg), or, SCC adjuvant alone as controls, in the presence or absence of simultaneous intraperitoneal administration of CD8^+^CD28^−^ or CD8^+^CD28^+^ T cells (4×10^5^ cells per mouse). Intraperitoneal abscess formation was examined at autopsy after 6 days. Each dot represents the abscess diameter per mouse; bars indicate the median abscess size per group; *p<0.05. B. Sp1-induced CD8^+^CD28^−^ T cells suppress CD4^+^ T cell proliferation *in vitro*. MLR was performed with DCs from Balb/c mice and CFSE-labeled CD4^+^ T cells from C57BL/6 mice in the presence or absence of Sp1-induced CD8^+^CD28^−^ or CD8^+^CD28^+^ T cells from C57BL/6 mice. Proliferation of CFSE-labeled CD4^+^ T cells was evaluated by flow cytometry. The upper histogram shows the result of the incubation of DCs with CFSE-labeled CD4^+^ T cells for 1 h (control), the lower histograms show the results after 5 days of incubation. The histograms show one representative experiment out of 3 assays performed with cells from three mice each. The percentage of CFSE-positive cells that exhibit proliferation between day 0 and day 5 is given above the marker (±SD; n = 3).

To address the function of Sp1-induced CD8^+^CD28^−^ T cells, we investigated their effect on CD4^+^ T cell responses. First, we examined the effect of CD8^+^CD28^−^ T cells on ZPS-induced abscess formation. In contrast to the adoptive transfer of Sp1-induced CD8^+^CD28^+^ T cells that together cause intraperitoneal abscesses with a median abscess size of 4 mm, transfer of Sp1-induced CD8^+^CD28^−^ T lymphocytes significantly inhibited the abscess formation (no abscess detected) (Fig. 3A). The intraperitoneal application of Sp1, as a positive control, induced abscesses with a median abscess size of 4 mm (one abscess/mouse), and the application of SCC alone and SCC plus mSp1 as negative controls, did not induce abscess formation. Transfer of CD8^+^CD28^−^ T lymphocytes from naïve mice also suppressed abscess formation (data not shown).

We also addressed the functional role of Sp1-induced CD8^+^CD28^−^ T lymphocytes in a mixed leukocyte reaction. DCs and CD4^+^ T cells of different haplotypes were co-incubated to provoke a mixed leukocyte reaction (MLR). Proliferation of the CD4^+^ T lymphocytes was visualized by their CFSE staining and flow cytometry. Fig. 3B demonstrates that in the absence of CD8^+^ T cells a proliferative response of CD4^+^ T lymphocytes takes place. When CD8^+^CD28^+^ T cells are added, CD4^+^ T lymphocytes proliferate to a similar degree. However, the co-incubation of DCs and CD4^+^ T cells in the presence of CD8^+^CD28^−^ T lymphocytes results in an efficient reduction of CD4^+^ T cell proliferation that is comparable to the control with non-proliferating CFSE-labeled CD4^+^ T cells measured on day 0 of incubation. These investigations show that Sp1-induced CD8^+^CD28^−^ T lymphocytes efficiently suppress a Sp1-mediated CD4^+^ T cell-dependent immune response *in vivo* and a strong non-antigen-specific CD4^+^ T cell response *in vitro*.

### Induction of CD8^+^CD28^−^ T lymphocytes is independent of direct APC/T cell contact and cytokines secreted by APCs

**Figure 4:**
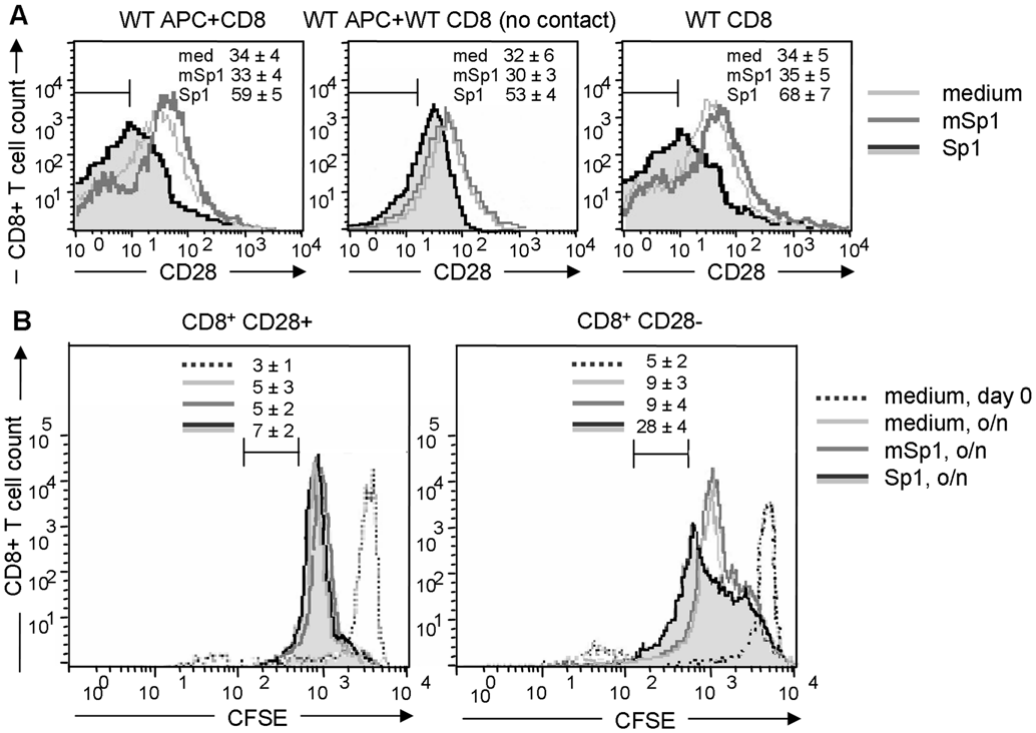
Sp1 induces CD8^+^CD28^−^ T lymphocytes *in vitro*. A. Total spleen cells from C57BL/6 WT mice, purified CD8^+^ T cells from WT mice separated by a transwell from WT spleen cells to prevent direct contact, and purified CD8^+^ T cells alone were treated for 16 h *in vitro* with Sp1 (100 µg/ml), mSp1 (100 µg/ml), or medium alone (left panel). Cells were stained for CD8 and CD28 surface expression and prepared for flow cytometry. The figure shows one representative histogram out of three assays performed with cells from 5 animals each. Percentages ± SD of the respective CD8^+^CD28^−^ cells are given in the upper right corner. B. CFSE-labeled CD8^+^ T cells were analyzed immediately after CFSE-labeling (medium, day 0), or incubated with Sp1, mSp1 (100 µg/ml, respectively), and medium overnight (o/n) and then analyzed for their proliferation capacity by flow cytometry. The figure shows one representative result out of three experiments performed independently with cells from 3 mice. The numbers above the marker in the histogram provides the percentage ±SD of T cells undergoing division for more than three generations, respectively.

To address the mechanism of the induction of CD8^+^CD28^−^ T cells by the Sp1 antigen, we first investigated the induction of CD8^+^CD28^−^ T lymphocytes by Sp1 *in vitro*. We stimulated spleen cells of C57BL/6 WT mice with Sp1, and mSp1 or medium alone as controls *in vitro*, and quantified the CD8^+^CD28^−^ population by flow cytometry. In comparison to medium- and mSp1-treated spleen cells of which 34% or 33% are of the CD8^+^CD28^−^ phenotype, respectively, 59% of spleen cells stimulated with Sp1 are characterized as CD8^+^CD28^−^ T cells (Fig. 4A). To investigate whether direct contact between APC and CD8^+^ T cells is required for the induction of CD8^+^CD28^−^ T cells, purified CD8^+^ T cells separated from spleen cells via a membrane with a pore size that did not allow APC/T cell contact, were treated with Sp1, mSp1, and medium. In contrast to medium- or mSp1-treated cells, Sp1 treatment induced an increase of the CD8^+^CD28^−^ T cell population in 21% and 23% respectively. This result left open the question, do cytokines secreted by the APCs induce CD8^+^CD28^−^ Ts? We therefore performed an assay with purified CD8^+^ T cells. 34% and 35% of CD8^+^ T lymphocytes stimulated with medium alone or mSp1, respectively, are CD28^−^, whereas 68% of the Sp1-treated purified CD8^+^ T cells are of the CD28^−^ phenotype. This result indicates that Sp1-mediated induction of CD8^+^CD28^−^ T lymphocytes is independent of direct contact between CD8^+^ T cells with APCs. Finally, we demonstrate that the induction of CD8^+^CD28^−^ T lymphocytes relies on expansion of this cell population (Fig. 4B). CFSE-labeled CD8^+^ T cells were incubated with Sp1, mSp1 and medium as controls, overnight and analyzed for their proliferation capacity. Fig. 4B demonstrates that in contrast to 7% of CD8^+^CD28^+^ T cells, 28% of CD8^+^CD28^−^ T cells undergo cell division more than three times in response to Sp1.

### Sp1 induces the proliferation of CD8^+^CD28^−^ T cells via TCR signaling

**Figure 5:**
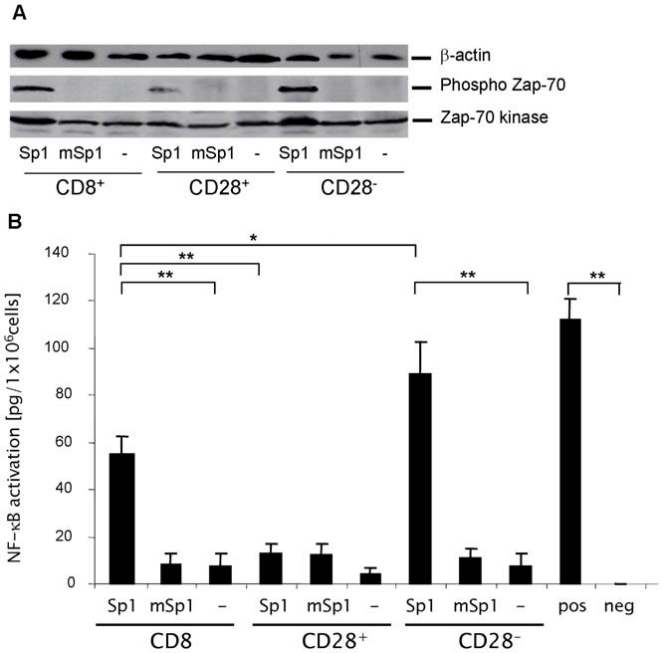
Sp1 induces TCR-mediated activation in CD8^+^CD28^−^ T lymphocytes. A. Sp1 induces phosphorylation of Zap-70 kinase and total Zap-70 kinase in CD8^+^CD28^−^ T lymphocytes. CD8^+^, CD8^+^CD28^−^, and CD8^+^CD28^+^ were incubated in the presence of Sp1, mSp1 (100 µg/ml, respectively), or in medium alone for 4 h. Western blotting was performed with a phospho-Zap-70, Zap-70 kinase-, and a β-actin-specific antibody as a control. A representative blot out of three experiments is shown. B. Sp1 induces NF-κB activity in CD8^+^CD28^−^ T lymphocytes. CD8^+^, CD8^+^CD28^−^, and CD8^+^CD28^+^ were incubated in the presence of Sp1, mSp1 (100 µg/ml, respectively), or in medium alone for 2 h. The nuclear extract of treated cells from 4 mice was analyzed by ELISA for NF-kB activity. As a positive control, the nuclear extract from Jurkat cells and as a negative control complete lysis buffer was used. The experiment was repeated three times in an independent manner. Columns indicate the mean NF-kB activity, bars indicate the standard deviation. **p<0.001,*p = 0.01.

Proliferation of T cells involves the engagement of TCR by an antigen. We investigated whether CD8^+^CD28^−^ T lymphocyte activation by Sp1 involves the TCR signaling cascade, resulting in T cell activation and proliferation. Following TCR engagement by an antigen, Zap-70 is rapidly phosphorylated which in turn results in enhanced Zap-70 kinase activity and downstream signaling events ultimately leading to NF-κB activation. We therefore incubated CD8^+^, CD8^+^CD28^−^, and CD8^+^CD28^+^ T cells in the presence of Sp1, mSp1 or medium alone and analyzed Zap-70 phosphorylation by western blotting. As shown in Fig. 5A, in contrast to medium alone or incubation with mSp1, treatment with Sp1 results in increased phosphorylation of Zap-70 in CD8^+^ T cells. Analysis of the CD28^−^ and CD28^+^ subpopulations revealed that CD8^+^CD28^−^ T lymphocytes are significantly more activated by Sp1 than CD8^+^CD28^+^ T lymphocytes. We next investigated NF-κB translocation activation. In correlation with the Zap-70 phosphorylation, Sp1 leads to significant NF-κB activation in CD8^+^ and CD8^+^CD28^−^ T lymphocytes, but not in CD8^+^CD28^+^ T cells (Fig. 5B). It is important to note that CD8^+^ T cells treated with mSp1 and PBS as controls were not activated.

### Sp1 prevents apoptosis and cell death of CD8^+^CD28^−^ T lymphocytes

**Figure 6:**
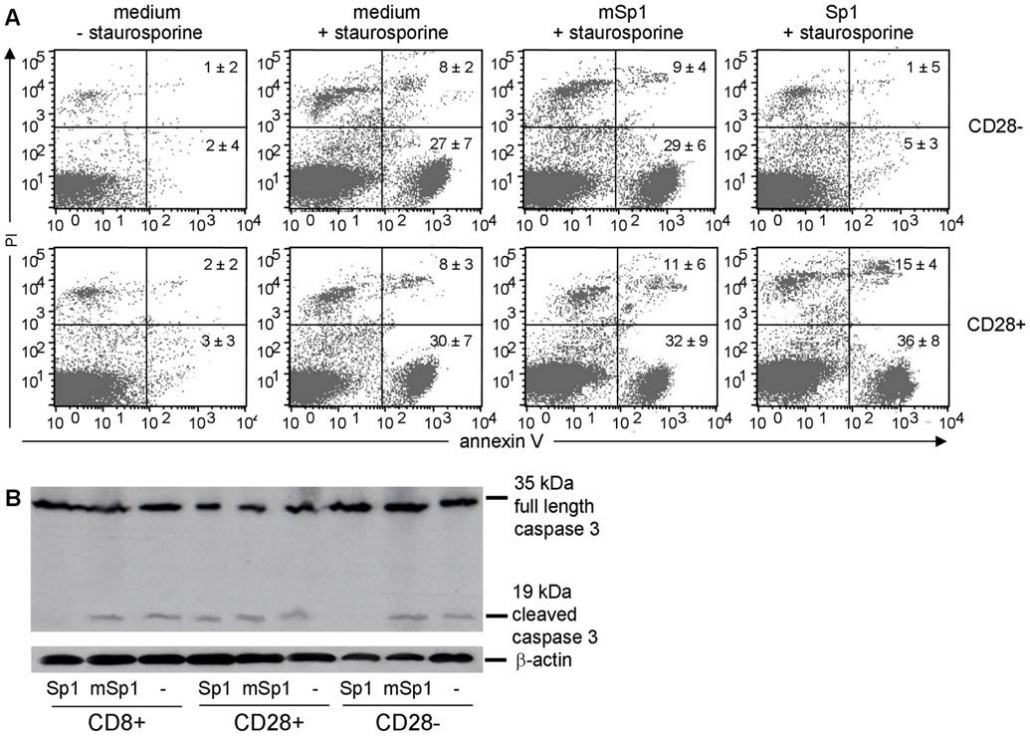
Sp1 prevents CD8^+^CD28^−^ T lymphocytes from apoptosis. A. CD8^+^ T cells from C57BL/6 spleens were treated in medium in the absence or presence of staurosporine with Sp1, mSp1 (100 µg/ml, respectively), or without antigen for 12 h. Magnetically separated CD8^+^CD28^−^ and CD8^+^CD28^+^ cells were stained with FITC-conjugated Annexin V and 5 µl propidium iodide (PI), and prepared for flow cytometry. One representative result out of three stainings with cells from 3 mice, respectively, is displayed. The numbers in the quadrants of the dot plots represent the percentage ±SD (n = 3) of cells stained positive for the respective markers. B. Sp1 inhibits staurosporine-induced caspase 3 activity in CD8^+^CD28^−^ T cells. CD8^+^, CD8^+^CD28^−^, and CD8^+^CD28^+^ T cells were treated for 4 h with mSp1, mSp1 (100 µg/ml, respectively), or in medium alone. SDS-PAGE of the whole cell extract was performed. One representative out of four western blots performed with a caspase 3- and a β-actin-specific antibody as a control is shown.

One of the central features of TCR-induced NF-κB action is to promote the expression of several anti-apoptotic proteins which enable cells to survive and to proliferate. To examine whether Sp1-induced T cell activation simultaneously prevents apoptosis, we treated CD8^+^ T cells with polysaccharide antigens in the presence or absence of staurosporine, a potent initiator of apoptosis [48]. We performed FACS analyses of CD8^+^CD28^−^ and CD8^+^CD28^+^ T lymphocytes and examined the extracellular exposure of annexin V as an indicator for apoptosis, and the uptake of the cell death marker propidium iodide (PI). Fig. 6A demonstrates that CD8^+^CD28^−^ T lymphocytes stimulated with Sp1 are protected from apoptosis and apoptosis-mediated cell death induced by staurosporine. Within the CD8^+^CD28^−^ T cell population stimulated with Sp1, only 5% of cells were apoptotic, and 1% were double-positive for the apoptosis and cell death marker - similar to the cells without staurosporine treatment. In contrast, non- and mSp1-treated CD28^−^ T cells are significantly susceptible to staurosporine-mediated apoptosis and cell death. 27% and 28% of CD8^+^CD28^−^ T cells, respectively, stained positive for annexin V and 8% and 9%, respectively, stained double-positive for annexin V and PI. Strikingly, Sp1 treatment does not confer any anti-apoptotic effect in the CD8^+^CD28^+^ T cell population. In this population, the percentage of apoptotic cells was similar to Sp1-, mSp1-, and non-treated cells (36%, 30%, and 32%, respectively). The same is true for the apoptosis and cell death double-positive cells (12%, 8%, and 11%, respectively). Furthermore, the anti-aoptotic effect of Sp1 was demonstrated by examination of executioner caspase 3 activation in CD8^+^CD28^−^ T cells treated with staurosporine. In resting and non-apoptotic cells caspase 3 is a p32 proenzyme which is proteolytically processed in apoptotic cells and forms an active p19 mature enzyme. The irreversible proteolytic processing of caspase 3 serves as an indicator for ongoing apoptotic process. These analyses underscore the anti-apoptotic potential of Sp1 and significantly demonstrated that Sp1 treatment specifically inhibits staurosporine-induced caspase 3 activity in CD8^+^CD28^−^ T cells but not in CD8^+^CD28^+^ T cells (Fig. 6B). In summary, we show by these analyses that Sp1 induces the activation and expansion of CD8^+^CD28^−^ T cells via TCR engagement and down-stream signaling which eventually results in inhibition of apoptosis in CD8^+^CD28^−^ T cells.

### Sp1 enhances TCR crosslinking

**Figure 7:**
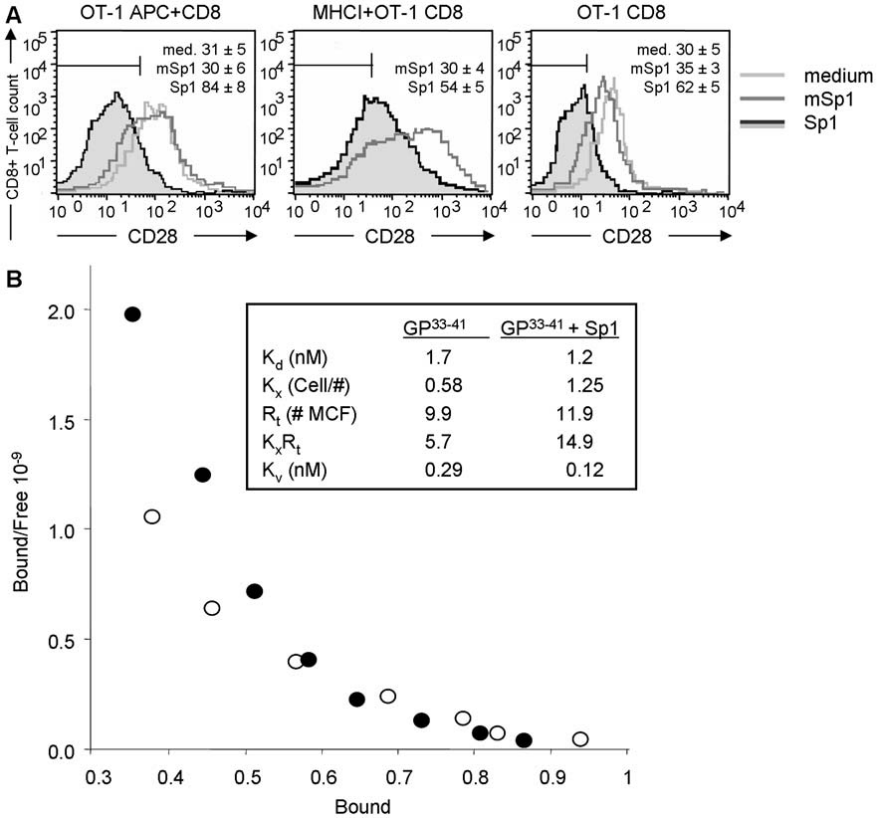
Sp1 induces peptide-specific CD8^+^CD28^−^ T lymphocytes and enhances TCR cross-linking on CD8^+^ T cells. A. Sp1 induces peptide-specific CD8^+^CD28^−^ T lymphocytes. Total spleen cells from OT-1 mice and purified OT-1 CD8^+^ T cells alone (purity >95%) were treated with SIINFEKL (10^−9^ M). SIINFEKL-loaded MHC class I dimers were coincubated with purified OT-1 CD8^+^ T cells. Cells were treated for 16 h *in vitro* with Sp1 (100 µg/ml), mSp1 (100 µg/ml), or medium alone. Cells were stained for CD8 and CD28 surface expression and prepared for flow cytometry. The percentages (±SD; n = 4) of the respective CD8^+^CD28^−^ cells are given in one representative histogram out of three experiments performed independently. B. Sp1 enhances TCR cross-linking on CD8^+^ T cells. CD8^+^ T cells from P14 mice were incubated with FITC-labeled and GP^33–41^- or ASN-loaded (nonspecific control) Db-Ig dimers in different concentrations in the presence or absence of Sp1. The mean fluorescence intensity was measured by flow cytometry. Using ASN as a control for nonspecific binding, the specific binding of GP^33–41^ (open circle) and GP^33–41^ plus Sp1 (filled circles) was calculated as described. A scatchard plot of the specific binding data is shown. The model fit parameters are summarized in the upper right box. K_d_ is the single site equilibrium dissociation constant. K_x_ is the equilibrium cross-linking constant with units of inverse number for receptors per cell. Rt is the total number of receptors available for binding in mean channel fluorescence (MCF) unit equivalents. K_v_ is the avidity at low dimer concentrations, which is estimated as K_v_∼K_d_/K_x_R_t_.

Activation of CD8^+^CD28^−^ T lymphocytes is mediated through TCR signaling. To test whether the induction of the CD8^+^CD28^−^ T cell population depends on peptide specificity and TCR specificity, we incubated spleen cells from OT-1 mice carrying a transgenic TCR on their CD8^+^ T cell surface for the specific recognition of the ovalbumin sequence SIINFEKL with Sp1, mSp1, or medium alone as controls, in the presence of the SIINFEKL peptide (10^−9^ M). Fig. 7A demonstrates that irrespective of the TCR specificity, Sp1 induces an increase of the CD28^−^ T cell population within the SIINFEKL-stimulated CD8^+^ T lymphocytes (84% versus 30% and 31% of medium- and mSp1-treated cells, respectively). We further analyzed Ts induction of CD8^+^ T cells with an artificial APC system that lacks APCs but contains MHC class I molecules loaded with SIINFEKL peptide. Compared to the negative control, Sp1 induced an increased expression in the CD28^−^ phenotype in 24% of CD8^+^ T cells. We also tested whether the induction of the CD8^+^CD28^−^ T cells requires MHC class I molecules at all and cultured purified CD8^+^ OT-1 T cells in the presence of SIINFEKL in medium alone, and in medium containing mSp1 or Sp1. Compared to the medium and mSp1 control, Sp1-treated CD8^+^ T cells show an CD8^+^CD28^−^ T cell increase of 32% and 27%, respectively. These results demonstrate an APC- and MHC class I-independent induction of the CD28^−^ phenotype by Sp1 in peptide-specific CD8^+^ T cells. At the same time, they allowed us to study the effect of Sp1 on the TCR membrane organisation using a MHC-Ig dimer model. Quantification of the TCR membrane organisation was achieved by fitting the binding data into a model of equilibrium dimerization of homogeneous monovalent receptors by divalent ligand [49] (Fig. 7B; upper right box). In this model, the first monovalent binding binds with single site affinity [50]. If there is another receptor nearby then the dimer may bind with both “arms” creating an “apparent binding affinity,” i.e., the avidity. Since the receptors are identical, increases in avidity can be attributed to increases in local concentration of receptors. In this model the single site dissociation constant, Kd, characterizes the binding of one site on the MHC to one TCR and the dimensionless cross-linking constant, K_x_Rt, characterizes the ability of the MHC/TCR complex to recruit another TCR. The binding avidity, K_v_, is approximately the ratio K_d_/K_x_R_t_ [51]. When there is minimal cross-linking potential, K_x_R_t_ is close to 1 and K_d_ and K_v_ are similar. If the cross linking potential K_x_ is high, there is an enhancement of binding due to dimerization, resulting in a stronger measured avidity than the intrinsic single site affinity. Analysis of the binding data indicated that the overall avidity, K_v_, of the dimeric MHC in the presence of Sp1 was ∼2.5-fold higher than in the absence of Sp1 (Fig. 7B). The increased avidity of MHC-Ig binding is due to an increased cross-linking constant (K_x_ = 0.58 cells/# in the absence of Sp1 vs. K_x_ = 1.25 cells in the presence of Sp1), rather than an increased intrinsic dissociation constant (K_d_ = 1.7 nM vs. K_d_ = 1.2 nM), or to changes in the total amount of receptors (R_t_ = 9.9 # mean channel fluorescence (MCF) vs. R_t_ = 11.9 # MCF). Thus, the enhanced avidity results from an increase in the cross-linking potential in the presence of Sp1.

## Discussion

In this paper we describe the first ever identification of a bacterial antigen that regulates CD4^+^ T cell immune responses through CD8^+^ T cells. A model ZPS antigen of commensal bacteria Sp1 induces CD8^+^CD28^−^ T lymphocytes which exhibit a suppressive function on CD4^+^ T cells in intraperitoneal abscess formation and in an allogeneic CD4^+^ T cell proliferation assay.

In the experimental model of abscess induction, CD8^+^CD28^−^ Ts represent a small population with about 0.1% of cells migrating into the peritoneal cavity upon Sp1 challenge. A portion of these cells is finally incorporated into the abscess wall. Although there is a small number of Ts cells participating in abscess formation, their depletion results in a significant enlargement of the abscess size, suggesting that they have an important role in their ability to regulate CD4^+^ T cell responses. We provide evidence that the Sp1-induced CD8^+^CD28^−^ T cells indeed act as suppressor cells. A regulatory/suppressive function of CD8^+^CD28^−^ Ts has been demonstrated in various *in vivo* experimental models such as, experimental autoimmune encephalitis and inflammatory bowel disease [34],[52]. However, this is the first ever demonstration that they can efficiently prevent intra-abdominal abscess formation. Abscess formation associated with secondary peritonitis, which is the most frequent type of peritonitis, causes severe morbidity and is often fatal with a mortality rate up to 30% [53],[54]. Even with optimal therapy including the administration of antibiotics and surgical treatment, residual abscesses form in many patients resulting in substantial morbidity and mortality [55]. Combining the cited reports and the result presented here, strongly suggest that CD8^+^CD28^−^ Ts cells play an important role in the physiological control of not only intestinal and cerebral, but also, of peritoneal immune responses.

Intraperitoneal application of Sp1 not only leads to an influx of CD8^+^CD28^−^ T cells into the peritoneum but also to an increase of these cells in the spleen, demonstrating a systemic effect of Sp1 on the immune system. In contrast, mSp1 without the positive charged substituent and therefore resembling a common negatively charged polysaccharide not only fails to induce CD4^+^ T cell-dependent abscesses but also CD8^+^CD28^−^ T cells in the peritoneum and in the spleen. Phenotype analysis of the Sp1-induced CD8^+^ T cells distinguishes them from the previously reported “naturally” inhibitory CD8^+^CD122^+^ T cell population [24],[25]. The CD8^+^CD28^−^ T lymphocytes induced by Sp1 express CD122 at a low level whereas the CD8^+^CD122^+^ population described in these reports correspond to CD8^+^CD122^high^ cells. Furthermore, it appears that the CD28^+^CD122^high^ identified in our study are CD44^high^CD62L^high^ thus classifying them as central memory cells [56]. The Sp1-induced CD8^+^CD28^−^ Ts cells are phenotype CD44^low^CD25^low^CD62L^high^ which categorizes them as naïve T cells, thereby, again clearly distinguishing them from CD8^+^CD122^+^ T cells. In conclusion, the induction of CD8^+^CD28^−^ T lymphocytes by Sp1 reflects an expansion of “naturally” CD8^+^CD28^−^ naïve T cells that had been isolated in spleens from naive C57BL/6 mice [34].

It is currently not understood whether the different types of regulatory CD8^+^ T cells described are distinct or overlapping cell populations because different markers have been employed for their characterization [28]. Given the need for a more coherent and broader phenotyping we expanded the description of the Sp1-induced CD8^+^CD28^−^ T cells to the surface markers CTLA-4 and CD39. In contrast to the CD8^+^CD28^+^ T lymphocytes, CD8^+^CD28^−^ T cells are CTLA-4^high^. CTLA-4 interacts with CD80 and CD86 expressed by APCs and by effector T cells to suppress T cell activation [57],[58]. CD4^+^CD25^+^ regulatory T cells inhibit T cell activation of effector cells *in vitro*. Interaction of CTLA-4 with CD80/CD86 is the only mechanism known to be involved in this regulatory process [59]. It is important to further study how and at what stage the CTLA-4 suppressor function intervenes in regulating immunity.

In the immune system, extracellular ATP exhibits multiple pro-inflammatory effects that can be inactivated by the ectoenzyme CD39 (nucleoside triphosphate diphosphohydrolase-1). The catalytic activity of CD39 is strongly enhanced by T cell receptor ligation [59],[60]. It was recently shown that CD39 is expressed primarily by immunosuppressive regulatory CD4^+^ T cells and that CD39, together with CD73, efficiently distinguishes T regulatory cells from other resting or activated T cells in mice [46],[59],[60]. Here, we observe a CD39^high^ phenotype on Sp1-induced CD8^+^CD28^−^ Ts. This result suggests that CD39 may not only be a marker on regulatory CD4^+^ but also on CD8^+^ Ts.

It has been suggested that IL-10 protects against inflammation in numerous *in vivo* and *in vitro* systems [61],[62]. A distinct population of CD4^+^CD25^−^ T cells produces IL-10 in response to the ZPS Sp1 and is responsible for protection in an experimental model of surgical fibrosis [14]. Here, we observe that ex CD8^+^CD28^−^ T lymphocytes stimulated *in vivo* with Sp1 also synthesize IL-10. We conclude that Sp1-induced CD8^+^CD28^−^ Ts belong to the group of naturally occurring CD8^+^ Ts that exhibit their regulatory function through IL-10 [24],[25],[26],[34],[39],[63],[64],[65]. TGF-β blocks T cell proliferation, Th1, and Th2 differentiation [47]. Our data show that the Sp1-induced Ts express TGF-β1 (as assessed by analysis of cell surface LAP). TGF-β has been described to be secreted by various Ts subpopulations: a) naturally occurring CD8^+^CD28^−^ Ts that prevented colitis in an experimental IBD model, b) induced CD8^+^ T cells that were generated by myelin basic protein and protected animals from EAE, c) CD8^+^CD25^+^Foxp3^+^ that inhibited proliferation of responder T cells *in vitro*, and d) CD8^+^Foxp3^+^ that prevented lupus [34],[66],[67],[68],[69],[70],[71],[72]. Most of these TGF-β-secreting Ts also suppressed T and B cell responses *in vitro*. Sp1-induced CD8^+^CD28^−^ Ts-derived IL-10 and TGF-β may not only contribute in controlling peritoneal immunity but additionally, modulate other inflammatory immune responses. It will be important to evaluate the precise mechanisms involved in the IL-10- and TGF-β-dependent prevention of intraperitoneal abscess formation, and other inflammatory processes of the host by Sp1-induced CD8^+^CD28^−^ regulatory T cells, because, regulatory lymphocytes are an attractive target for the treatment of various inflammatory processes.

The ZPS PS A2 from *B. fragilis* and Sp1 from *S. pneumoniae* are known to form a wide right-handed α-helix structure with the positively charged groups turned towards the outside of the molecule allowing facultative interactions with protein molecules or glycoproteins [6],[7]. MHC class II molecules have open-ended antigen binding grooves [41] and therefore can accommodate antigen molecules that are larger than the groove, such as, the 15 kDa-size PS A1 or Sp1 saccharide. MHC class I has a closed antigen binding groove and therefore it is unlikely that a ZPS fragment is presented by MHC class I. We tested this hypothesis and demonstrated that the expansion of CD8^+^CD28^−^ Ts is independent of direct contact of APC and T cell, and the MHC class I molecule. Consequently, activation of CD8^+^CD28^−^ T cells due to presentation of Sp1 in the MHC class I binding groove or cross linkage of MHC and TCR - similar to the activation mechanism employed by superantigens – is excluded. Furthermore, APC-mediated NF-kB activation of T cells through TCR has been shown to be dependent on the engagement of CD28 co-receptor by its ligands B7-1 (CD80) and B7-2 (CD86) on APCs [73]. However, Sp1 is capable to initiate NF-kB activation in CD8^+^ T cells lacking CD28 co-receptor. Therefore, it is conceivable that Sp1 because of its pronounced repetitive and charged structure binds externally to T cell surface molecules such as the TCR that mediate an activation signaling cascade. We demonstrate that Sp1 predominantly induces Zap-70 phosphorylation of the CD8^+^CD28^−^ subpopulation and ultimately leads to their NF-kB activation. Consequently, this cell population is protected from cell death. Several factors countering cell death cascades have been shown to be positively regulated by NF-kB activation. Inhibition of mitochondrial apoptotic cascade in CD4^+^CD28^−^ T cells isolated from patients with rheumatoid arthritis has been shown to be the underlying molecular mechanism which gives rise to expansion and accumulation of CD4^+^CD28^−^ T cells in these patients [74]. Here in CD8^+^CD28^−^ T cells, Sp1 confers resistance to the broad range kinase inhibitor staurosporine which preferentially initiates mitochondrial apoptotic cascade [48]. Thus, Sp1 treatment promotes NF-kB activation in CD8^+^CD28^−^ T cells which leads to blockade of mitochondrial apoptotic function. However, the nature of this specific modification of mitochondrial apoptotic machinery in CD8^+^CD28^−^ T cells by Sp1-treatment remains to be determined.

The proliferation of Ts does not depend on the specificity of its TCR which enabled us to approach investigations of the mechanism of TCR activation. Dimeric MHC-Ig complexes have been used as potential tools for studying T cell membrane organizations [50],[75]. By means of Db-Ig MHC-Ig dimers we calculated the affinity, avidity, and the numbers of receptors on GP^33–41^-specific T cells, in the presence and absence of Sp1 during antigen-specific binding. Since we did not see any relevant changes regarding the affinity or numbers of the receptors, but a significant increase in the avidity, we conclude that Sp1 exerts its T cell activation via an enhanced cross-linking of TCRs. Data from *in vitro* induced CD8^+^CD28^−^ T cells after priming with specific antigens showed that the CD8^+^CD28^−^ Ts did not respond to the priming antigens [29]. Our data indicate that Sp1 modulates CD8^+^ T cell responses induced by different specific peptides through non-peptide- and non-TCR-specific enhancement of the TCR cross-linking. T cell responsiveness to an epitope is affected both by its affinity for the presenting MHC molecule as well as by the affinity between the MHC-peptide complex and the TCR. Low affinity interactions may result in weak T cell responses [76]. In this respect, ZPSs may enhance the stability and crosslinking of the SMAC and subsequently trigger CD8^+^ T cell immune functions, including suppressive properties of CD8^+^CD28^−^ T cells. On the other hand, since avidity changes in the TCR represent a method of increasing the sensitivity of activated T cells it is also possible that ZPS-mediated cross-linking of the TCR sensitizes antigen-specific CD8^+^ cells, including CD8^+^CD28^−^ suppressor cells, to lower levels of antigen [50]. We propose the following model regarding the mechanism of Ts induction by ZPS: ZPS crosslinks TCR molecules on CD8^+^CD28^−^ T lymphocytes that are engaged in recognizing and binding to specific antigens of foreign or self origin, and, lead to their activation, proliferation, cytokine secretion and non-antigen-specific suppressive function on T cells. Interestingly, proliferation of Ts does not depend on the specificity of its TCR but clearly depends on the presence of the positive charge on the polysaccharide molecule. This observation implies that the zwitterionic charge that determines the three-dimensional α-helix configuration is the dominant feature in TCR-mediated T cell activation.

Taken together, these data explain survival and expansion of the CD8^+^CD28^−^ population. But, we do not yet have an answer to the question, why, almost exclusively the CD8^+^CD28^−^ and not the CD8^+^CD28^+^ become activated by Sp1. Various scenarios are possible and will need to be addressed in the future. 1. The lack of expression of the CD28 co-stimulatory surface molecule might promote better binding of Sp1 to the TCR due to less steric hindrance. 2. The lack of expression of the CD28 molecule on the T cell surface is associated with a phenotype and functionally unique cell biology. Consequently, other molecules that remain to be identified may be as relevant as the CD28 protein for the susceptibility of CD8^+^CD28^−^ T cell activation by Sp1. 3. Post-translational modifications such as glycosylation of proteins play a key role in immune regulation [77],[78]. Changes of the glycosylation state, mainly desialysation have been shown to enhance class I MHC-TCR interactions and T cell stimulation [78]. ZPSs are repetitive highly charged molecules. It seems conceivable that binding of ZPS enhances cross-linking of TCRs due to different charge states of the TCR or co-receptors on CD28^−^ and CD28^+^ T cells. The enhanced cross-linking of TCR caused by a polysaccharide antigen represents an exciting novel mechanism in cellular immunology. Further investigations will be needed to explore in detail, the interaction of ZPS with the TCR of CD8^+^ effector and regulatory T cells.

In conclusion, ZPS from commensal bacteria represent a unique class of antigens that exhibit multiple functions on different host cells and contribute to the maintenance of peripheral tolerance. Here we demonstrate that the ZPS Sp1 induces regulatory CD8^+^ T cells inhibiting CD4^+^ T cell immune responses *in vitro* and *in vivo*. The Sp1-induced CD8^+^CD28^−^ Ts increase the knowledge of physiologic bacterial antigens and with it, the possibility to develop cell-based therapies against unwanted immune responses in human beings.

## Materials and Methods

### Ethics statement

Animal experiments were performed in accordance with the German animal protection legislation guidelines (article K07/05 and K16.5/06) and approved by the “Bezirksregierung Köln”, Germany.

### Antigens

*S. pneumoniae* type 1 capsular polysaccharide complex was obtained from the American Type Culture Collection and further purified to obtain homogeneity as previously described [10]. Chemical modification of Sp1 by neutralization of the free amino group on the 2-acetamido-4-amino-2,4,6-trideoxygalactose by N-acetylation that creates a polysaccharide with a net negative charge was performed as previously described [1]. The capsular polysaccharide of group B streptococcus serotype III (GBSIII) was kindly provided by Dennis L. Kasper. High-resolution (500 MHz) proton NMR spectroscopy [7] revealed that Sp1 and modified Sp1 (mSp1) were free of contaminating protein and nucleic acids. Endotoxin was not detectable by the limulus test with a sensitivity of <8 pg LPS/mg Sp1. The peptides ASN (sequence H-ASNENMETM-OH) and GP^33–41^ (sequence H-KAVYNSATM-OH) were obtained from Mimotopes, the SIINFEKL (sequence H-SIINFEKL-OH) from JPT.

### Mice

Wildtype C57BL/6 mice were obtained from Charles River Laboratories. OT-1 C57BL/6 mice expressing a transgenic receptor specific for the ovalbumin peptide sequence SIINFEKL were obtained from Jackson Laboratories. P14 TCR-tg C57BL/6 (P14) mice expressing a transgenic receptor for the GP^33–41^ peptide sequence of the lymphocytic choriomeningitis virus were generously provided by J. P. Schneck [79]. Mice were kept in special pathogen-free environments.

### Experimental model of abscess formation

In the abscess induction studies, 6–8 week-old mice were injected intraperitoneally with Sp1 or modified Sp1 (mSp1) (100 µg in PBS mixed with sterile cecal content (SCC) adjuvant; 1∶1 v/v, 0.2 ml total volume) or SCCA alone, as control [10]. SCC is a required steril adjuvant in this model and mimics leakage of the intestinal flora during secondary peritonitis [1]. For the preparation of SCC, cecal content of non-treated mice is grinded through 100 µm-pore size meshes and autoclaved thereafter. The SCC dose not able to induce abscesses when applied alone is determined by titration in the experimental model of abscess formation. In order to block CD4^+^ or CD8^+^ T cells intraperitoneally, 24 h prior to challenge, mice were injected intravenously with a CD4-specific mAb (clone YTS 169, 500 µg per mouse) or a CD8-specific mAb (clone YTS 191, 500 µg per mouse), or left untreated. Depletion of CD4^+^ T cells and CD8^+^ T cells was confirmed by flow cytometry analysis of whole blood and spleen cells 24 h and 48 h after injection. Analysis showed depletion of >95% of the CD4^+^ and CD8^+^ T cell population (data not shown). For adoptive transfer studies, CD8^+^CD28^−^ or CD8^+^CD28^+^ T cells (4×10^5^ per mouse) were injected intraperitoneally at the time of polysaccharide challenge. Six days after challenge, mice were macroscopically examined for the presence of abscesses within the peritoneal cavity by two double-blinded examiners. Abscesses were isolated and their diameter measured. In each experiment, four to six mice per group were tested. The experiments were performed three times in an independent manner.

### Immunohistochemistry

Snap-frozen abscesses were cryo-sectioned (5–6 µm), fixed in 4% buffered formalin for 1 min and then used for immunohistochemical analysis of CD8^+^ T cells. After blocking endogenous peroxidase by 0.3% H_2_O_2_ in methanol, and endogenous biotin with an avidin-biotin blocking kit (Vector Laboratories) for 30 min, sections were treated with normal goat serum then incubated overnight with an anti-CD8 antibody (1∶50; rat anti-mouse CD8, clone Ly-2, BD). Next, goat biotin-conjugated anti-rat antibodies (1∶100, BD) were applied and incubated for 1 h at room temperature followed by incubation with alkaline phosphatase-conjugated streptavidin-Ab complex (Dako) for 30 min. Immunostaining was achieved using Fast Red (Dako) as a substrate for alkaline phosphatase. Sections were then counterstained with hemalaun.

### Cells and cell culture

Peritoneal cells were obtained at different times following intraperitoneal Sp1, mSp1, or PBS challenges. Mice underwent peritoneal lavage with 4 ml of ice-cold RPMI-1640 supplemented with 10% FBS and 1% penicillin/streptomycin. Total cell count was obtained with a hemocytometer after trypan blue staining. Each sample was then analyzed by flow cytometry.

CD4^+^ and CD8^+^ T cells were enriched from spleens of C57BL/6 mice by, grinding the tissue through a 70 µm mesh, lysis of red blood cells, centrifuging in Ficoll-Hypaque gradients (density 1,083), and, immunomagnetic negative selection according to the manufacturer's instructions (Miltenyi Biotec, R&D). The purity of the cell population was confirmed by flow cytometry (>95%).

CD8^+^CD28^−^ and CD8^+^CD28^+^ T cells were isolated as follows. CD8^+^ T cells were incubated with PE-labeled anti-CD28 mAb for 20 min, washed, centrifuged, and incubated with anti-PE-microbeads for 15 min at 4°C (Miltenyi). Alternatively, CD8^+^ T cells were incubated with a biotinylated anti-CD28 mAb for 10 min at 4°C, washed, centrifuged, and labeled with streptavidin-APC for 15 min at 4°C. PE- or APC-labeled CD28^+^ cells were enriched by positive selection using anti-PE- or anti-APC-labeled microbeads, respectively (Miltenyi). Magnetic negative selection of CD28^−^ was performed following the manufacturer's instructions (Miltenyi). For adoptive transfer studies of CD28^−^ and CD28^+^ T cells, CD8^+^ T lymphocytes were stained with FITC-labeled anti-CD8 and PE-labeled anti-CD28 for 20 min on ice. CD8^+^CD28^−^ and CD8^+^CD28^+^ T lymphocytes were sorted electronically using a FACSVantage (BD) More than 93% purity was routinely obtained (Fig. S1).

DCs were generated from mouse bone marrow by adapting a previously described method [80]. In brief, bone marrow cells from C57BL/6 or Balb/c mice were cultured in RPMI-1640 supplemented with 5% FBS, 500 U recombinant mouse granulocyte/macrophage-colony stimulating factor (GM-CSF), 20 µg/ml gentamicin, and 50 µM 2-mercaptoethanol. The DC medium was exchanged at two-day intervals. DCs were isolated by magnetic cell sorting with a CD11c-specific mAb (Miltenyi Biotec).

Experiments involving overnight culturing of WT CD8^+^ T cells were performed in RPMI-1640 supplemented with 1% L-glutamine, sodium pyruvate, penicillin-streptomycin, non-essential amino-acids, 50 µM 2-ME, 10% FBS (Life Technologies), and purified anti-CD3 mAb (5 ng/ml). Cell culture with abrogated contact between WT APCs and T cells were performed with the CD8^+^ T cells in the upper chamber of transwell inserts with a 0.4 µm pore size (Corning) and spleen cells in the lower chamber. In experiments with OT-1 CD8^+^ T cells, instead of purified anti-CD3 mAb, SIINFEKL was used at a concentration of 10^−9^ M. Co-cultering of purified CD8^+^ T cells from OT-1 mice with SIINFEKL-loaded H2K^b^ dimers was performed as followed: MHC class I-Ig dimer (Kb-Ig; BD) were loaded with peptide by incubation in a 40-fold molar excess of the specific peptide SIINFEKL as instructed by the manufacturer. 5×10^5^ purified CD8^+^ T cells from OT-1 mice were incubated with SIINFEKL-loaded MHC class I-Ig dimers (8 µg) in the absence or presence of Sp1 (100 µg/ml) in 200 µl culture medium at 37°C overnight

### Flow cytometry and intracellular cytokine staining

For staining surface markers, cells were stained with specific antibodies for 30 min on ice, washed, and then analyzed by flow cytometry.

To analyze the cellular intraperitoneal influx, the absolute number of each respective cell type present in the peritoneal lavage was calculated by taking the proportion of each cell type as determined by flow cytometry and multiplying it by the total number of cells per ml of lavage obtained from each mouse.

For intracellular cytokine staining (ICS), cells were stimulated with anti-CD3 (5 ng/ml) for 6 h at 37°C and 5% CO_2_. After the first hour of incubation, Golgi Stop (BD) was added. Cells were stained for surface markers by 30 min on ice with specific antibodies, washed, fixed with Cytofix/Cytoperm (BD) for 20 min on ice, then permeabilized with Perm/Wash Solution (BD) for 10 min on ice and treated with interleukin-specific antibodies for 30 min at 4°C in Perm/Wash Solution.

The apoptotic capability of Sp1-treated CD8^+^ T cells was analyzed after staurosporine treatment using Annexin V staining. 1×10^6^ CD8^+^ T cells/ml isolated from the spleens of C57BL/6 mice were incubated in medium (RPMI-1640 supplemented with 5% FCS) containing 1 µM staurosporine (*Streptomyces* sp., Calbiochem-Novabiochem) in the presence of Sp1, mSp1 (100 µg/ml, respectively), or in medium alone. Cells were washed after 4 h to free cells from staurosporine. Thereafter, cells were incubated in fresh medium plus antigens for another 8 h. As a control, medium without staurosporine was used. After washing, cells were dissolved in binding buffer (0.1 M Hepes/NaOH (pH 7.4), 1.4 M NaCl, 25 mM CaCl (BD)) and stained with FITC-conjugated Annexin V (5 µl/1×10^6^ cells; BD Apoptosis Detection Kit) for 15 min on ice. Cells were washed, and 5 µl propidium iodide (PI) (BD) was added to the cells for 2 min. The PI staining was stopped by the addition of 5 ml of ice-cold PBS. Cells were washed and prepared for flow cytometry.

Purified, FITC-, PE-, or APC-labeled monoclonal antibodies specific to murine CD8 (clone RM4-5, BD), CD25 (clone 7D4, BD), CD28 (clone 37.51, BD), CD44 (clone IM7, BD), CD62L (clone MEL-14, BD), CD69 (clone H1.2F3, BD), CD39 (clone TU66, BD), CD122 (clone TM-BETA 1, Serotec), LAP (clone 27232, R&D), CTLA-4 (clone UC10-4B9, eBioscience), and the respective isotype controls were used for surface marker staining. For ICS, mIL-6 (clone MP5-20F3, BD), anti-mouse mIL-10 (clone JES5-16E3, BD), and the respective isotype controls were used. Cells prepared for flow cytometry were analyzed – after gating for viable cells by forward and side scatter - with FACS Calibur™ (Becton Dickinson) using CELLQuest™ software (Becton Dickinson). The results were expressed as percentage (%) or mean fluorescence intensity (MFI) of fluorescence-labeled cells in a population. Experiments were performed a minimum of three times in an independent manner.

### Mixed leukocyte reaction

Allogeneic mixed leukocyte reaction (MLR) was performed as described previously [81]. DCs from Balb/c mice (H-2d haplotype) were incubated with negatively selected (Miltenyi) and CFSE-labeled CD4^+^ T cells of C57BL/6 mice (H-2b haplotype) (1.5×10^5^/well) in the absence or presence of CD8^+^CD28^−^ or CD8^+^CD28^+^ T cells isolated from C57BL/6 mice (5×10^5^/well) treated for 18 h with Sp1 (100 µg/ml). Cells were incubated for 5 days at 37°C, 5% CO_2_. Proliferation of CFSE-labeled CD4^+^ T cells was evaluated by flow cytometry.

### Western blotting

For the analyses of Zap-70 and caspase 3 activity, magnetically separated CD8^+^, CD8^+^CD28^−^ and CD8^+^CD28^+^ were incubated in medium (RPMI-1640 supplemented with 5% FCS) in the presence of Sp1, mSp1 (100 µg/ml, respectively), or in medium alone for 4 h at 37°C. For analysis of caspase 3 activity, cells were treated in medium containing 1 µM staurosporine. Whole cell extracts were denatured at 100°C for 10 minutes. Equal amounts of protein were separated by 14% Tris-glycine sodium dodecyl sulfate polyacrylamide gel electrophoresis (SDS-PAGE). Western blotting was performed with purified mouse anti-Phospho-Zap-70 (Tyr319)/Syk (Tyr352) (Cell Signaling Technology), mouse anti-Zap-70 kinase (clone 29/ZAP70 kinase, BD), rabbit anti-caspase 3 (clone 8G10, Cell Signaling Technology), anti-ß-actin (clone AC-15, Sigma Aldrich), goat anti-mouse IgG HRP, and goat anti-rabbit IgG HRP (R&D) antibodies as described previously [48].

### NF-κB Analysis

Magnetically separated CD8^+^, CD8^+^28^−^ and CD8^+^CD28^+^ T cells were treated with Sp1, mSp1 (100 µg/ml, respectively) or left untreated for 2 h at 37°C in medium (RPMI-1640 supplemented with 5% FCS) for 2 h. Cells were washed, counted, and incubated for 15 min in low salt buffer on ice before NP-40 was added. After sedimenting the nuclei, a high salt buffer was added slowly. The nuclear extract is separated from the nuclear envelop/DNA by centrifuging at maximum speed. A BCA assay was performed to determine the protein concentration. The NF-κB ELISA was performed according to the manufacturer's instructions (Active Motif). As positive control the nuclear extract from Jurkat cells was used.

### Dimer and binding assays

MHC class I-Ig Dimer (Db-Ig) were prepared as described previously [50],[82]. Db-Ig dimers were labeled with fluorescein isothiocyanate (FITC; Molecular Probes) at pH 7.4 and purified by gel column chromatography. Concentration of protein and dye was measured by assessing the absorbance at 280 nm and 496 nm respectively. For loading, FITC-labeled Db-Ig dimers were incubated in a 50-fold molar excess of the specific peptide GP^33–41^ and a two fold-molar excess of human β_2_-microglobulin for five days at 4°C. ASN was used as a control peptide to address nonspecific binding. For binding assays, CD8^+^ T cells were isolated from P14 mice. 1×10^5^ cells were incubated with varying amounts of protein-loaded Db-Ig dimers in the presence or absence of Sp1 (100 µg/ml) at 4°C in darkness for 90 min. Without any washing stages, binding was measured by flow cytometry. Subtraction of the nonspecific binding (^ASN^Db-Ig) from the total binding (^GP33–41^Db-Ig) determined the specific binding. The single site equilibrium dissociation constant (K_d_), the equilibrium cross-linking constant (K_x_) and the total number of receptors available for binding (R_t_) were calculated using Microcal Origin 6.1 software (Origin Lab Corporation, Northampton, USA) [50].

### Statistical analysis

Results of peritoneal cytokine and cellular influx assays in the various groups were compared by Student's t-test. Comparison of groups with regard to abscess formation was made by chi-square analysis. Results of NF-κB ELISA were compared by Student's t-test.

## Supporting Information

Figure S1 Purity of CD8^+^CD28^−^ and CD8^+^CD28^−^ T cells. CD28^−^ and CD28^+^ CD8^+^ cells for adoptive transfer studies were sorted by flow cytometry as described in the Materials and Methods section and evaluated for their purity.
(0.02 MB PDF)
