# Supplementary figures and images for: Streptococcus pneumoniae Serotype 1 Capsular Polysaccharide Induces CD8+CD28− Regulatory T Lymphocytes by TCR Crosslinking
Source: PLoS Pathog. 2009 Sep 25;5(9):e1000596. doi: 10.1371/journal.ppat.1000596 (PMC2742891; doi:10.1371/journal.ppat.1000596)

Suppl. Figure S1, Mertens et al.

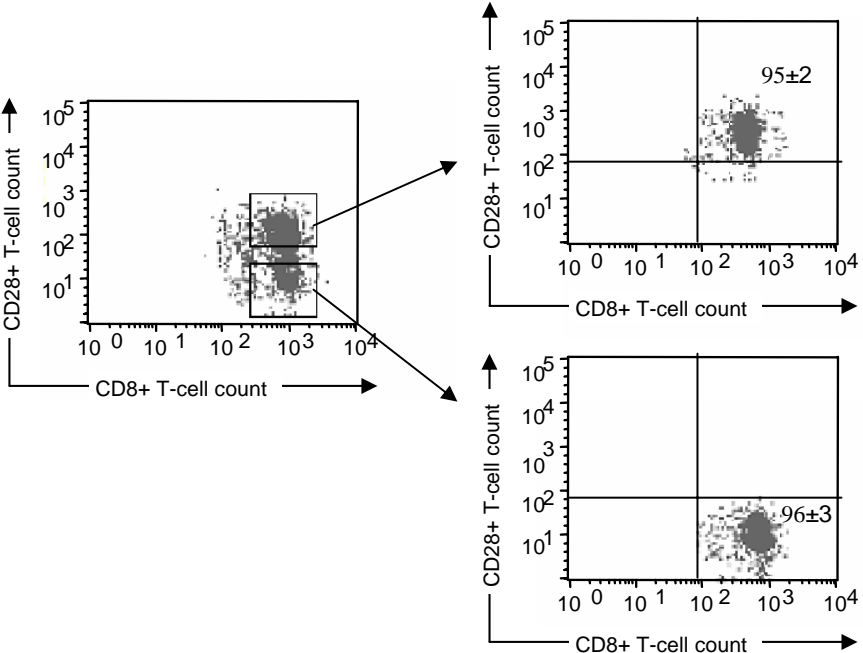

Supplement: Figure S1 — Purity of CD8+CD28− and CD8+CD28− T cells. CD28− and CD28+ CD8+ cells for adoptive transfer studies were sorted by flow cytometry as described in the Materials and Methods section and evaluated for their purity. (0.02 MB PDF) [file ppat.1000596.s001.pdf]
